# Microbiological Characteristics of Some Stations of Moscow Subway

**DOI:** 10.3390/biology11020170

**Published:** 2022-01-21

**Authors:** Andrei A. Pochtovyi, Daria V. Vasina, Bakhtiyar I. Verdiev, Alexey M. Shchetinin, Anton G. Yuzhakov, Roman S. Ovchinnikov, Artem P. Tkachuk, Vladimir A. Gushchin, Alexander L. Gintsburg

**Affiliations:** 1 Federal State Budget Institution “National Research Centre for Epidemiology and Microbiology Named after Honorary Academician N F Gamaleya” of the Ministry of Health of the Russian Federation, 123098 Moscow, Russia; d.v.vasina@gmail.com (D.V.V.); yuryevpolskei@yandex.ru (B.I.V.); schetinin.am@gmail.com (A.M.S.); anton_oskol@mail.ru (A.G.Y.); rsovchinnikov@mail.ru (R.S.O.); artem.p.tkachuk@gmail.com (A.P.T.); gintsburg@gamaleya.org (A.L.G.); 2 Department of Virology, Biological Faculty, Lomonosov Moscow State University, 119991 Moscow, Russia; 3 Laboratory of Biochemistry and Molecular Biology, Federal State Budget Scientific Institution “Federal Scientific Center VIEV”, 109428 Moscow, Russia; 4 Laboratory of Mycology and Antibiotics, Federal Research Center “All-Russian Research Institute of Experimental Veterinary Medicine (VIEV) Named after K.I. Skryabin and Y.R. Kovalenko“ of Russian Academy of Science, 109428 Moscow, Russia; 5 Department of Infectiology and Virology, Federal State Autonomous Educational Institution of Higher Education I M Sechenov, First Moscow State Medical University of the Ministry of Health of the Russian Federation (Sechenov University), 119435 Moscow, Russia

**Keywords:** microbiome, subway, 16S rRNA gene, aerosol, surface, AMR, public health

## Abstract

**Simple Summary:**

Public transport facilities, including subway systems, provide the most suitable conditions for the transfer of microorganisms between people and the environment, contributing to the pathogenic potential of the urban habitat. Investigation of microbiome diversity and description of its characteristic properties, e.g., antibiotic-resistance profiles, leads to understanding of these interactions. In this study, we aimed to conduct an extended analysis of the bioaerosol and surface microbiome of the Moscow subway, using 16S rRNA gene sample sequencing and classical microbiology methods. The microbiomes of two subway stations (Novokosino and Cherkizovskaya) were reconstructed which differ in terms of passenger traffic and duration of exploitation. It was shown that most bacterial genera were ubiquitous; however, the unique genera were presented in aerosol samples. The relatively older Cherkizovskaya station possessed greater diversity in antibiotic resistance among the identified microorganisms compared to Novokosino station. We also provided a comparative analysis of these results with the previously published data, which allowed us to identify the distribution of microorganisms associated with the human microbiome and the environment regardless of the seasonal fluctuations. The obtained results provide valuable information on the diversity of bacterial communities in the Moscow subway, one of the most socially important facilities in metropolitan areas.

**Abstract:**

The subway is one of the most actively used means of transport in the traffic infrastructure of large metropolitan areas. More than seven million passengers use the Moscow subway every day, which promotes the exchange of microorganisms between people and the surrounding subway environment. In this research, a study of the bacterial communities of two Moscow subway stations was conducted and the common subway microbiome was determined. However, there were differences in microbiological and antibiotic-resistance profiles, depending on the station. The station’s operational period since opening correlated with the taxonomic diversity and resistance of the identified bacteria. Moreover, differences between aerosol and surface bacterial communities were found at the two subway stations, indicating the importance of diversified sampling during the microbiome profiling of public areas. In this study, we also compared our data with previously published results obtained for the Moscow subway. Despite sample collection at different stations and seasons, we showed the presence of 15 common genera forming the core microbiome of the Moscow subway, which represents human commensal species, as well as widespread microorganisms from the surrounding environment.

## 1. Introduction

The study of the diversity of microorganisms in the air and on various surfaces of socially significant objects is gaining in importance with every year and is an essential task aimed at ensuring biological safety and preventing the spread of infectious diseases. Public transport systems, such as subways, airports, and train stations, provide the most suitable conditions for pathogen transfer between people. The subway has recently become the most favored form of public transport in large cities, used daily by up to 50% of the population. The population of Moscow and its agglomeration is 17 million people, and the passenger traffic of the subway exceeds 7.016 million passengers a day [1], which makes the Moscow subway one of the largest in the world and the largest in Europe [2]. Opening in 1935, the Moscow subway today comprises 14 lines and 250 stations, which are located in various economic and geographical areas of Moscow. The number of passengers depends on the line, station, and time, with higher passenger traffic at terminal stations and transfer stations. Terminal stations connect the city center with the densely populated residential areas, where the majority live. The Moscow subway has two characteristic peaks of passenger traffic that are primarily associated with a working schedule: in the morning (approximately from 07:30 to 09:00) and evening (from 18:00 to 19:30). At the same time, the average travel distance of an individual passenger in the subway is 15–20 km. These numbers correspond approximately to a 30–40 min time interval spent in the subway. Considering the emission of about 10^6^ particles per hour in the air exhaled by one person [3,4], a total of 8.18 × 10^12^ particles are excreted per day, thereby forming a significant component of the microbiome of the air and surfaces in the subway.

Since many microorganisms are capable of aerosol transmission and various particles from surfaces can be transferred to a suspended state as a result of airflow, bioaerosol monitoring is an important task. Due to the lack of a unified bioaerosol collection method, most microorganism diversity studies in the subway concentrate on a simpler and more coherent protocol of sample collection from various surfaces, whereas bioaerosol diversity in combination with surface microbiome assessments have been carried out only for some subways [5].

Various surfaces in four stations of the Moscow subway were characterized in an earlier study [6]. However, this study was a pilot and did not include an assessment of viability or pathogenicity, according to the authors.

The purpose of our study was to conduct an extended analysis of the microbiomes of bioaerosol and various surfaces in the Moscow subway. At the same time, our attention was focused on two contrasting stations. We studied changes in the microbiome depending on the type of sample (air aerosol or surface) and the level of passenger traffic. Combining variable regions of 16S rRNA gene sequencing with classical microbiology methods made it possible to characterize differences in microbiomes and evaluate microorganisms that were resistant to antibacterial drugs.

## 2. Materials and Methods

### 2.1. The Study and Sampling Site

Air and surface samples were collected in the checkpoint areas of two Moscow subway stations in February 2017. Two contrasting stations were selected to allow the comparison and demonstration of changes in microbiomes. First of all, these stations varied with respect to passenger traffic, Novokosino with 76,000 passengers/day and Cherkizovskaya with 24,000 passengers/day. Additional characteristics of the selected stations are presented in Table S1. Collection of material was carried out in two stages. Each stage included three consistent samplings of 15 min duration each. The first stage started with the departure of the first train and lasted from 06:00 to 06:45. The second stage occurred at the time of the passenger traffic increase, from 08:00 to 08:45.

### 2.2. Air Sampling with Air Sampler SASS 2300 and a Virtual Impactor SASS 4000—Method for Aerosol Assessments

The SASS collection method was carried out with a combination of the SASS 2300 sampler (Research International Inc., Monroe, WA, USA) and a virtual SASS 4000 impactor (Aerosol) with a set flow rate of 4000 L/min, with a concentration of 0.5–10 μm collected particles in 4.5 mL sterile PBS solution. The SASS 4000 virtual impactor was installed at a height of 150 cm. Collected samples were stored and transported in a refrigerator on ice at −20 °C before they were delivered to the laboratory. The obtained samples were divided into 1 mL aliquots and deposited at −80 °C. Each 1 mL volume aliquot was centrifuged at 7000× *g* for 20 min to precipitate microorganisms. The SASS was washed for 8 min between two time slots. For the SASS aerosol sampler, internal flushing without aerosol sampling was used as a negative control.

### 2.3. Surface Swabs Sampling—Method for Surface Assessments

Surface samples were collected using sterile viscose swabs LOQSwabs (Copan Diagnostics, Murrieta, CA, USA). Each swab had been previously soaked in a sterile PBS solution, and samples were taken from surfaces with which people had been in contact recently, namely, turnstiles and checkout areas. The collection area was 30 cm^2^. Clean swabs were used for negative controls.

### 2.4. Microbiological Analysis

Selected samples were cultured on the following solid medium for microbiological testing: Columbia Blood Agar medium, LB medium, Baird-Parker medium, Endo agar, and *Enterococcus* agar (HiMedia Laboratories Pvt., Mumbai, India). Transfer of liquid samples onto solid medium was carried out in a volume of 0.1 mL per Petri dish. The suspension was evenly distributed on the surface of a solid medium with a sterile L-shaped spatula. Plates were incubated at a temperature of 37 °C for 48 h. Colonies were observed on each solid medium after an appropriate incubation period, and their morphology was described.

After cultivation, the most typical colonies (morphotypes) were classified with the use of MALDI-TOF mass spectrometry. In brief, a single bacterial colony was resuspended in 150 μL of sterile deionized water. Then, 350 μL of 96% ethanol was added to each sample, which was vortexed and centrifuged for 2 min at 8000× *g* and 4 °C. The supernatant was removed, and the washing step was repeated twice. Forty microliters of a 70% formic acid solution were added to each sample. Subsequently, 40 μL of 99% acetonitrile was added and the solution was vortexed and centrifuged for 2 min at 8000× *g* and 4 °C. One microliter of each sample in triplicate was placed on a metal plate and left to dry in a sterile chamber at room temperature. Then, 1 μL of α-cyano-4-hydroxycinnamic acid (10 mg/mL) was applied to each spot of the sample and left to dry. The plate was analyzed with a MALDI-TOF mass spectrometer MALDI Biotyper (Bruker Scientific LLC, Billerica, MA, USA) using the MALDI Biotyper 3.0 software package (Bruker Scientific LLC, Billerica, MA, USA). Scores above 1.699 implied reliable identification of the genus and probable species identification.

### 2.5. Resistance Determination by Direct Sample Transfer to Selective Medium

For this study, we pooled samples based on the collection method, station, and time. To achieve this goal, we took 300 μL of the initial sample, merged it into 1.5 mL Eppendorf tubes, and gently mixed. Determination of bacterial resistance was carried out by 100 μL of a liquid sample transfer onto a solid Mueller Hinton nutrient medium containing one of the antibiotics: tetracycline (10 mcg/mL), streptomycin (20 mcg/mL), ampicillin (100 mcg/mL), kanamycin (50 mcg/mL), chloramphenicol (30 mcg/mL), and gentamicin (10 mcg/mL). Antibiotic solutions were prepared immediately before nutrient medium preparation. The Mueller Hinton medium was sterilized, cooled to 50 °C, then solutions of selected antibiotics were added to the medium, and the obtained liquid was poured onto Petri dishes. The Mueller Hinton medium without antibiotic solution was used as a control. Cultivation was carried out at 37 °C for 48 h. After incubation, a quantitative analysis of the grown colonies was performed on all Petri dishes. Cultures grown on a medium with antibiotics were transplanted into separate plates, then samples for species identification were prepared using the MALDI-TOF method, as was described earlier.

### 2.6. DNA Isolation Targeted Amplification and Sequencing

To study the uncultivated metagenome, total DNA from aerosol and swab samples was isolated using a PureLink™ Microbiome DNA Purification Kit (Thermo Fisher Scientific, Waltham, MA, USA). Since the amount of DNA in the samples was low, the number of amplification cycles was increased to 30 according to the manufacturer’s instructions.

Amplification of the seven most variable regions of the 16S rRNA gene (V2, V3, V4, V6, V7, V8, and V9) was performed using the Ion 16S™ Metagenomics Kit (Thermo Fisher Scientific, Waltham, MA, USA). To test the amplification of the required specific regions, electrophoresis in 2% agarose gel was performed with visualization with ethidium bromide. A library cleaning purification was performed using Agencourt AMPure XP magnetic particles (Beckman Coulter, Brea, CA, USA). Amplicon barcoding was performed using IonCode Barcode Adapters (Thermo Fisher Scientific, Waltham, MA, USA), according to the manufacturer’s instructions. Sequencing was performed on an Ion S5XL sequencer (Thermo Fisher Scientific, Waltham, MA, USA) using Ion 520 and Ion 530 Chips (Thermo Fisher Scientific, Waltham, MA, USA). The sequence data were deposited in the NCBI Sequence Read Archive under accession number PRJNA788451.

### 2.7. Sequence Analysis

As a result of the sample preparation, sequences of seven variable regions of the 16S rRNA gene were obtained using the 16S Metagenomics Kit. Separation of reads into regions was carried out as described previously [7]. Analyses were conducted for six different subsets using the DADA2 package [8]. Denoising was used with some additions to match the one-way reads and error-aware Ion Torrent sequencing techniques (HOMOPOLYMER_GAP_PENALTY = −1, BAND_SIZE = 32). Chimeras were removed using the “consensus” method. Taxonomic assignment was performed using the DADA 2 package and the naive Bayesian classifier method with SILVA SSU v.132 [9] as the base software. The amplicon sequence variant (ASV) table, taxonomy table, and metadata were imported into the R package phyloseq for analyses [10]. The package Decontam in R was used to remove contaminating sequences using the “combined” method by comparing the prevalence of each ASV and frequency (concentration) in samples after processing the sequence data [8].

Further analysis was carried out only with samples rarefied to 31,006 reads. The Mann–Whitney test was performed to compare alpha diversity, with *p* < 0.05 considered statistically significant. Beta-diversity was estimated using Bray–Curtis dissimilarity with the permutational multivariate analysis of variance (PERMANOVA) test.

### 2.8. Comparative Analysis with Previously Published Data on the Study of the Microbiome of the Moscow Subway

For a comparative analysis of the microbiome, the variable region V4 of the 16S rRNA gene sequencing data was obtained from a previously conducted study (hereinafter referred to as Knomics [6]). The samples mentioned in this article were swabs from various surfaces of four stations. The research was performed in June 2016 (accession number in SRA: PRJNA495018). Taking into account differing primers and sequencing technologies, all sequences were normalized to common coordinates and total length. While normalizing, we were forced to trim two sequences: “CCAGCAGCCGCGGTAATACGT” from the 5′ end and “GGATTAGATACCCGTGGTAGTCC” from the 3′ end. In the Knomics data, “TACGG” and “AGG” were removed from the 5′ and 3′ ends, respectively. Taxonomic classification and subsequent various metrics calculations were carried out as was described earlier. The rarefaction was carried out with a depth of 2913 reads. The dplyr [11], UpSetR [12], ggplot2 [13], and ggpubr [14] packages were used to identify common genera and, in addition, to plot the graph.

## 3. Results

### 3.1. Sequencing Data Analysis

In this work we analyzed 24 samples collected at two subway stations. We obtained 11,180,826 reads related to seven variable regions of the 16S rRNA gene as a result of sequencing. The most number of reads was obtained for the V3 (3,618,249 reads) and V4 regions (2,248,597 reads). After a set of filtration stages, low-quality reads trimming, and contaminating sequences removal, the remaining sequences were assigned to 793 genera that belong to 289 different families.

### 3.2. Microbiome Analysis of Two Moscow Subway Stations

**Figure 1:**
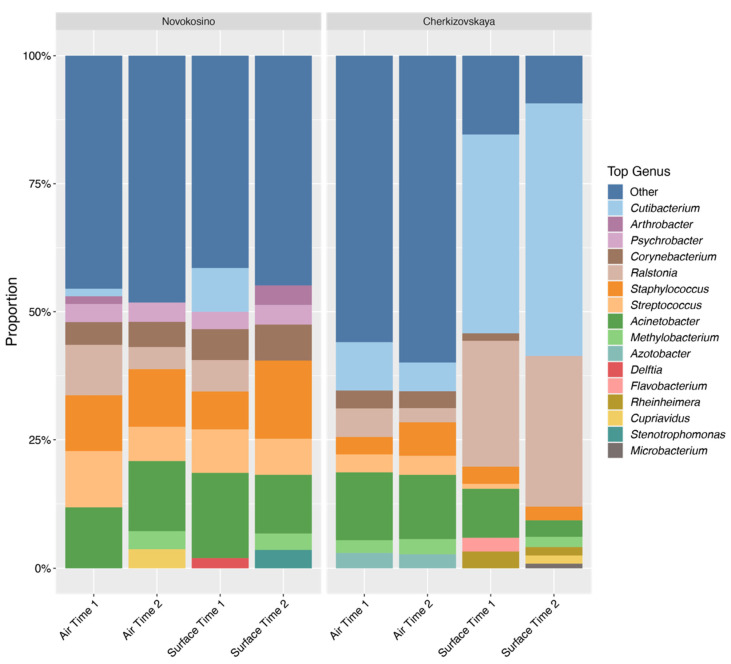
Changes in the representation of the most common genera, depending on the collection method and the station.

The taxonomic distribution at the genus level demonstrated the similarity between the aerosol and surface samples collected at Novokosino and Cherkizovskaya (Figure 1). The most common genera were represented by *Acinetobacter*, *Streptococcus*, *Staphylococcus*, and *Ralstonia*, which are widespread in the environment and are symbionts with human skin. Moreover, *Corynebacterium* and *Cutibacterium* were identified in almost all samples. There was an increase in the proportion of the genus *Methylobacterium* at Novokosino station with the traffic, regardless of the type of sample, while at Cherkizovskaya station its occurrence was mostly constant.

Among the most common genera at Novokosino station were *Arthrobacter*, *Psychrobacter*, *Delftia*, and *Stenotpophomonas*, which were not represented in the 10 most common genera at Cherkizovskaya station. An increase of passenger traffic at Novokosino station correlated with a change in the proportion of *Corynebacterium*, *Methylobacterium*, and *Staphylococcus* genera in aerosol and surface samples. Aerosol and surface samples were the most contrasting for *Cupriavidius* (predominant in the aerosol in the second time interval), *Delftia* (predominant on the surface in the first time interval), and *Stenotrophomonas* (predominant on the surface during the second time interval). For representatives of the genera *Cutibacterium* (which are considered as commensals of human skin and opportunistic microorganisms) and *Ralstonia* (opportunistic microorganisms widespread in the environment), the proportion increased in the second time interval, during the increase in passenger traffic, and these microorganisms were characteristic mostly for surfaces. The eight most represented genera of the Novokosino microbiome comprised about 50% of all sequenced microorganisms.

**Figure 2:**
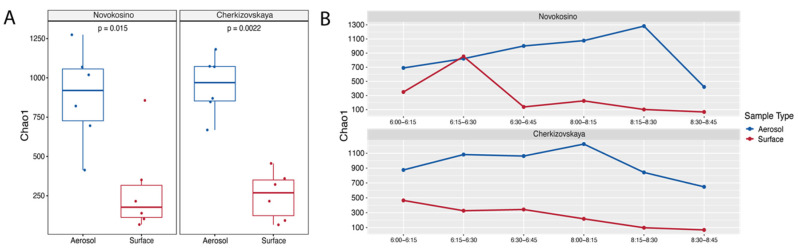
Comparison of alpha diversity with respect to the collection method and the station. (**A**) Diversity measured by the Chao1 index. Box plots with middle line denote the median, the box denotes the interquartile range (IQR), and 1.5 IQR ranges (whiskers). The Mann–Whitney test was performed to compare alpha diversity with *p* < 0.05 considered statistically significant. (**B**) Dynamics of the Chao1 index change depending on the collection method and the station. Red—aerosol (SASS collection method), blue—surface (swab collection method).

The taxonomic profile of the aerosol and surface samples was much more diverse at Cherkizovskaya station. In addition to common genera, its microbiome was characterized by the presence of *Azotobacter* in aerosol and *Rheinheimera*, *Flavobacterium*, and *Microbacterium* in surface-collected samples. The surface microbiome at Cherkizovskaya station became increasingly scarce over time with the dominance of *Cutibacterium* and *Rheinheimera* genera, the proportion of which increased from 39.2% and 24.3% to 49.6% and 29.3%, respectively, and was associated with an increase in passenger traffic. At the same time, the percentage of less well represented genera that were merged into the “Other” group decreased over time from 15.2% to 9.1%. We did not note the predominance of any genera in the aerosol samples. At the same time, the diversity was great regardless of the station and time of collection. This can be seen from the alpha diversity assessment data (Figure 2).

### 3.3. Diversity

It was found that the Chao1 indexes of alpha diversity are significantly higher for the microbiome of aerosol samples (Figure 2A). In addition, the Mann–Whitney test (*p* < 0.05) confirmed significant differences in microbiome composition depending on the origin of the sample and the station of collection. Taking into account the dynamics of a microbiome assessed according to the Chao1 index, its change over time in correspondence with the growth of passenger traffic can be indicated (Figure 2B). In general, the Chao1 index showed higher values for the aerosol samples, thereby allowing a more accurate assessment of diversity variations. For Novokosino station, it continuously grew from the beginning of the research (Chao1 index = 690) and reached its maximum in the interval from 08:15 to 08:30 (Chao1 index = 1288). For Cherkizovskaya station, there was an increase from 06:00 to 06:30, where the Chao1 index values reached a plateau and equaled 1062–1082. The maximum value of the Chao1 index was noted at 08:00–08:15 and equaled 1223. Samples from surfaces were generally characterized by a lower Chao1 index, which varied in the range of 67–852 at Novokosino station and in the range of 68.6–648 at Cherkizovskaya station.

**Figure 3:**
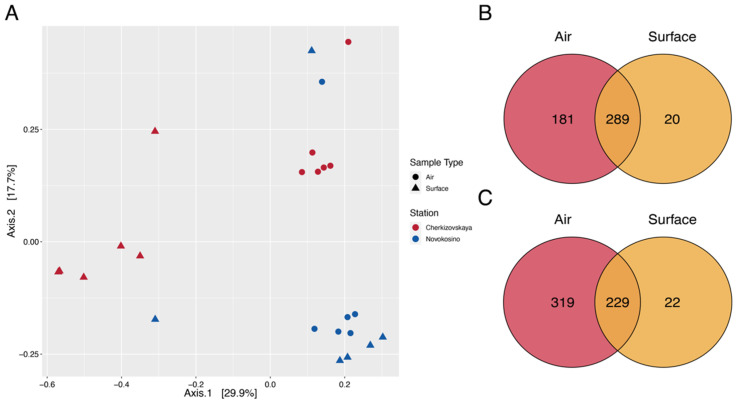
(**A**) Bray–Curtis dissimilarity PCoA was used to generate ordination of beta-diversity in Station (color) and Method (shape). Principal coordinates 1 and 2 (Axis 1 and Axis 2) explained 29.9% and 17.7% of the variance in the Bray–Curtis dissimilarity. (**B**) The distribution of ASV across aerosol and surface samples for Novokosino station. (**C**) The distribution of ASV across aerosol and surface samples for Cherkizovskaya station.

Principal coordinate analysis (PCoA) was conducted with the use of the Bray–Curtis dissimilarity matrix to evaluate clustering (and potential separation) of samples depending on the type and the station. We found three main clusters: two clusters characterized separate stations and one cluster was common for two stations (Figure 3A). We found significant differences in the multivariate PERMANOVA model with predictors such as Station and Method (F = 5.8883, R^2^ = 0.18165, *p* = 0.0001; and F = 5.4318, R^2^ = 0.16757, *p* = 0.0001, respectively). The number of shared bacterial genera for aerosol and surface samples was 289 and 229 for Novokosino and Cherkizovskaya stations, respectively. Most of the unique genera (181 and 319) were identified for air aerosols. However, their proportion was around 2.37% and 5.89% of the total number of reads. In addition, 20 species from Novokosino station and 22 from Cherkizovskaya station were unique for the surface samples; their proportion was 0.27% and 0.14%, respectively (Figure 3B,C).

### 3.4. Determination of Cultivated Microbiome Morphotypes’ Resistance to Antibacterial Drugs

The cultivation of the obtained samples was carried out to assess the resistome profiles of the microorganisms. Twenty-seven morphotypes (MTs) were identified during cultivation. Among these colonies, 16 were identified according to species (Figure S1). Regardless of the time and collection method, *Micrococcus luteus* was predominant at Novokosino station. In addition, bacteria of the *Staphylococcus* genus (*S. saprophyticus*, *S. epidermidis*, and *S. warneri*), as well as *Aerococcus viridans* and *Acinetobacter schindleri*, were found in air samples. At the same time, the proportion of *M. luteus* and *Staphylococcus* spp. increased with an increase in passenger traffic (at the second time point of sample collection); in contrast, the representation of *A. viridans* decreased. The number of unidentifiable microorganisms was significantly higher in comparison to the Novokosino station samples (2180 CFU vs. 140 CFU).

**Figure 4:**
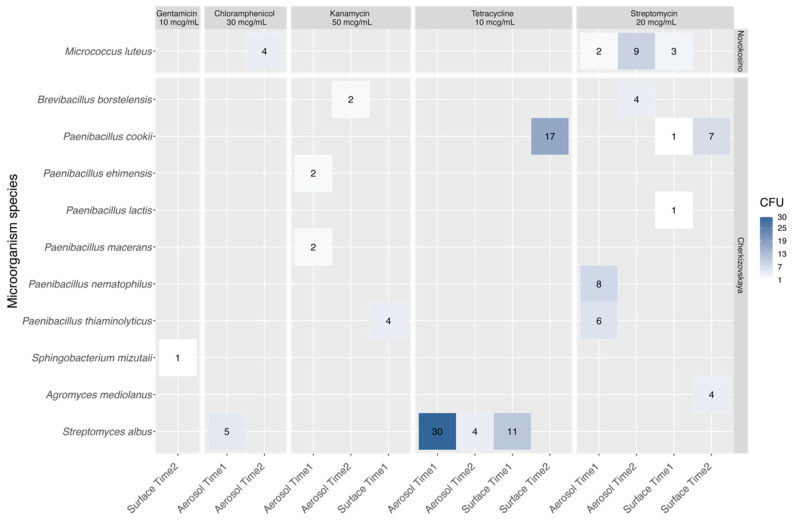
Heat map of resistance of various microorganisms to antimicrobial drugs. The numbers indicate the quantity of CFU.

Among the identified MTs, antibiotics resistance was determined (Figure 4, Table S2). Thus, the resistome of Novokosino station was represented with a single species, *Micrococcus luteus*, known as an opportunistic human pathogen. It was found to be resistant to streptomycin and chloramphenicol out of the six studied antibiotics. At the same time, cultures, resistant to chloramphenicol, were detected only in the second time interval, when passenger traffic was increased. Ampicillin resistance was not determined.

The Cherkizovskaya station possessed more diverse profiles of resistant bacteria. Among them, *Streptomyces albus* resistant to chloramphenicol and tetracycline was found in almost all experimental samples. Various representatives of the genus *Paenibacillus* were resistant to several antimicrobial agents, including kanamycin (*P. ehimensis* and *P. macerans*, collected by Aerosol Time 1), while the isolate of *Paenibacillus cookie* showed resistance to tetracycline (Surface Time 2), and *P. nematophilus* and *P. thiaminolyticus* (Aerosol Time 1), *P. lactis* (Surface Time 1), and *P. cookie* (Surface Time 1 and Surface Time 2) showed resistance to streptomycin. *Brevibacillus borstelensis* (Aerosol Time 2) was found to be resistant to kanamycin and streptomycin, and *Agromyces mediolanus* (Surface Time 2) also showed resistance to streptomycin. Only one isolate of *Sphingobacterium mizutani* was resistant to gentamicin.

### 3.5. Comparison with Previously Published Data

**Figure 5:**
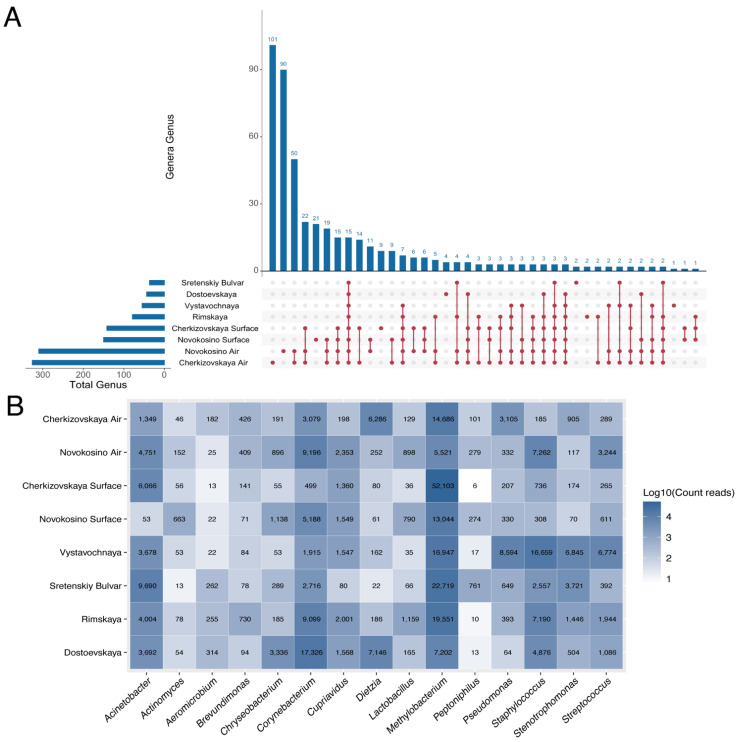
Common and unique bacterial genera according to two independent parts of the research. (**A**) An UpSet plot illustrates the common and unique microbial genera at the stations of the Moscow subway. The number on the left indicates the total number of genera in the sample, and the number above the bar indicates the number of unique/common genera for groups marked below the bars. (**B**) Heat map illustrates the representation of the 15 most common genera at all six stations. The numbers indicate the number of reads.

In addition, we conducted a comparative assessment with the previously studied microbiomes of Sretenskiy Bulvar, Dostoevskaya, Rimskaya, and Vystavochnaya stations. Comparative analysis revealed a large number of unique genera for Novokosino and Cherkizovskaya stations (Figure 5A). In total, 15 genera were common and presented both in the aerosol and on surfaces at all six stations (Figure 5B). *Corynebacterium*, *Staphylococcus*, *Stenotrophomonas*, *Streptococcus*, *Pseudomonas*, and *Acinetobacter* were among them. These representatives are widely distributed in people’s residences and are essential components of the human microbiome. Among the most represented unique genera (their proportion exceeds 5% of the number of reads) were the genus *Shuttleworthia* (19.95%), which was detected in the aerosol of Novokosino station; *Chromohalobacter* (11.76%), *Arcticibacter* (8.14%) and *Galbibacter* (7.83%) were detected at Cherkizovskaya station. For surfaces at Novokosino station, *Veillonella* (20.32%); Cherkizovskaya, *Brevibacillus* (21.74%), *Porphyrobacter* (14.13%), *Terrimonas* (10.8%), *Vitellibacter* (10.87%), and *Paraprevotella* (9.78%); Vystavochnaya, only one genus, *Alkaliphilus* (100%); Sretenskiy Bulvar, *Pelosinus* (75.0%) and *Anaerospora* (25.0%); Rimskaya, *Arsenicicoccus* (86.27%) and *Anaerocolumna* (13.73%); Dostoevskaya, *Tissierella* (26.45%), *Caproiciproducens* (25.61%), *Antricoccus* (23.96%), and *Pseudogracilibacillus* (23.96%).

## 4. Discussion

The Moscow subway is one of the key elements of the public transport system; it is an essential transport unit for the daily life of megapolis citizens. Heavy traffic and constant contact with aerosol and various surfaces increase the chances of infection transmission between passengers [5,15,16]. The microbiome study enables expansion of our knowledge about the diversity of microorganisms in aerosol and on surfaces circulating in public facilities, allowing us to take a deeper look at the dynamic changes of microorganisms and to evaluate their resistance to antimicrobial drugs. All of this contributes to a better understanding of the impact of these factors on human health.

In this study, we conducted metaprofiling and antimicrobial resistance characterization in aerosol and surface samples from two stations of the Moscow subway, which ranks as the fifth or sixth largest in the world and first in Europe in terms of passenger traffic.

The study of the taxonomic diversity of our samples demonstrated the aerosol and surface profiles at the stations, which corresponded with the data from other world subway systems. The majority of the identified genera (*Acinetobacter*, *Streptococcus*, *Streptococcus*, and *Corynebacterium*) are commensals of human skin and mucous membranes [6,17,18,19], which are widespread in the subways of different cities all around the world (New York, Oslo, Mexico City, Athens, and Boston) [17,18,20,21]. Some genera (*Cutibacterium*, *Ralstonia*, and *Psychrobacter*) were predominant either at the station as a whole or were mostly found in the aerosol or on the surfaces. It was shown that *Cutibacterium* was a predominant representative in the Mexico City subway [21]. *Ralstonia* was more characteristic for air aerosol samples in the Oslo subway. Previously, a higher concentration of the *Psychrobacter* genus was noted in the Oslo subway in winter [5]. Another widespread genus of the Moscow subway, *Methylobacterium*, was found to be dominant in the Barcelona subway [22] and was included in the top 23 highly distributed genera in the Athens subway [18]. Representatives of the genus *Methylobacterium* are aerobic microorganisms capable of growing in the presence of formaldehyde, formate, and methanol [23]. Thus, they act as biological indicators of pollution [22]. For both stations, we also found an increase in the proportion of the genus *Cupriavidus* with the growth of traffic, which in general may indicate an increased content of heavy metals in the environment [24,25].

Evaluation of the Chao1 diversity index revealed significant differences in the diversity of microorganisms depending on the type of sample (aerosol or surface) over time. We noted a more stable alteration of the Chao1 index for aerosol samples, while samples from surfaces had a more stochastic character and characterized the microbiome in the given time period. It can be assumed that the method of collection from surfaces does not reflect the real dynamics of microorganisms despite the simplicity of the collecting methodology and the possibility of protocol standardization. The obtained results demonstrate the greater efficiency of using the aerosol collection method compared with swabs from the surface during short-term sampling (15 min) for subsequent assessment of microorganism profile variability. Despite the different levels of passenger traffic, the majority of the cultivated microbiome was represented by skin commensals and microorganisms from the environment—*Micrococcus luteus*, *Aerococcus viridans*, *Staphylococcus epidermidis*, and *Staphylococcus saprophyticus* [26,27,28,29,30,31,32]. These species occurred in larger numbers in the surface samples compared to aerosol samples and were probably the result of direct skin peeling with subsequent precipitation and transfer to a suspended state in air aerosol. At the same time, the proportion of such bacteria as *Staphylococcus saprophyticus*, *Staphylococcus epidermidis*, and *Staphylococcus warneri* correlated with passenger traffic. Other representatives of the cultivated microbiome were widespread microorganisms, which are rarely evaluated as pathogens [30,33,34,35,36,37,38].

The study of the antibiotic resistance of the collected samples demonstrated a great difference between the two stations. There was only one resistant species (*Micrococcus luteus*) at Novokosino station and 10 species that demonstrated greater diversity and resistance to various groups of antibacterial drugs at Cherkizovskaya. Resistance to gentamicin (n = 1), chloramphenicol (n = 1), kanamycin (n = 4), tetracycline (n = 4), and streptomycin (n = 7) was shown among these species. Most species were resistant to two antibiotics (mainly, it was a combined resistance to streptomycin and kanamycin). A species of *Paenibacillus macerans*, which had resistance to three drugs at once—streptomycin, kanamycin, and chloramphenicol—was also isolated. In humans, single cases of infection with *Paenibacillus* sp. were associated with immunodeficiency or traumatic conditions [39,40]. In general, the detected microorganisms are considered as human symbionts, and their pathogenic potential is minimal.

Differences in microbiomes revealed between the stations can be explained by the duration of their exploitation, workload, the territorial location, and the materials used in the construction of the station. Cherkizovskaya station was opened in 1990 and has already formed its microbiome, while Novokosino station was opened relatively recently in 2012 and is actually still forming its stable microbiome.

A comparative analysis of the microbiome from samples collected in the Moscow subway based on the results of two studies made it possible to identify 15 genera found at all stations. Taking into account the collection of samples in February 2017 (our study) and in June 2016 (the Knomics study), it is possible to assume the presence of these genera regardless of the season, passenger traffic, and their ubiquity both in the aerosol and on the surface. Despite the constant core microbiome, we note the presence of subtle differences at the level of genera, which suggests a difference in the stations themselves and the possible influence of seasonal fluctuations. Among these were isolated representatives of *Tissierella* sp., an anaerobic sulfidogenic bacterium that has an affinity to metallic copper [41], *Caproiciproducens*, isolated from sewage treatment plants [42], and representatives of *Arsenicicoccus* capable of metabolizing arsenic from wastewater [43]. The characteristic features of the identified microorganisms propose the prevalence of the species adapted to survival in an urban setting. Most of the unique genera have been isolated from the environment (soil and water) and are barely etiological human pathogens. The microorganisms forming the microbiome of the Moscow subway stations are not significant etiological pathogens and are mostly natural inhabitants of urban infrastructures. They are essential for maintaining such an environment and represent a “normal” metagenomic profile of the city [44,45].

## 5. Conclusions

In this paper, we focused on two different stations of the Moscow subway and characterized the microbiome of their aerosol and surfaces comprehensively. The obtained data indicated the formation of a core microbiome at each station, depending on the characteristics of the station and the level of passenger traffic. It also identified the distribution of microorganisms associated with human microflora, which are not susceptible to seasonal fluctuations.

## Data Availability

The sequence data have been deposited in the NCBI Sequence Read Archive under accession number PRJNA737285.

## Supplementary Materials

The following supporting information can be downloaded at: https://www.mdpi.com/article/10.3390/biology11020170/s1, Table S1: Comparative characteristics of the studied stations; Figure S1: Heat map with identified morphotypes. The numbers indicate the number of CFU; Table S2: Detected antibiotic-resistant isolates. MALDI NRI—not reliable identification using MALDI Biotyper.
