# Supplementary material for: Microbiological Characteristics of Some Stations of Moscow Subway
Source: Biology (Basel). 2022 Jan 21;11(2):170. doi: 10.3390/biology11020170 (PMC8869165; doi:10.3390/biology11020170)
Supplement: Supplementary file 1 [file biology-11-00170-s001.zip › biology-1534211-supplementary.pdf]

Table S1. Comparative characteristics of the studied stations.

Station name	Novokosino	Cherkizovskaya
Line name (Color line)	Kalininskaya (yellow)	Sokol’nicheskaya (red)
Opening year	2012	1990
Station Depth, m	9	11
Passenger traffic, thousand people/day	76	24
Administrative division	Novokosino	Preobrazhenskoe
The population of the district, thousand people	107.9	91.5
Area Administrative division, km²	3,60	5,614
Population Density, personspeople/km²	29974.17	16299.07
Cost of housing, thousand rubles/m² ($/m²)	137.84 (2106.88)	191.72 (2995.62)

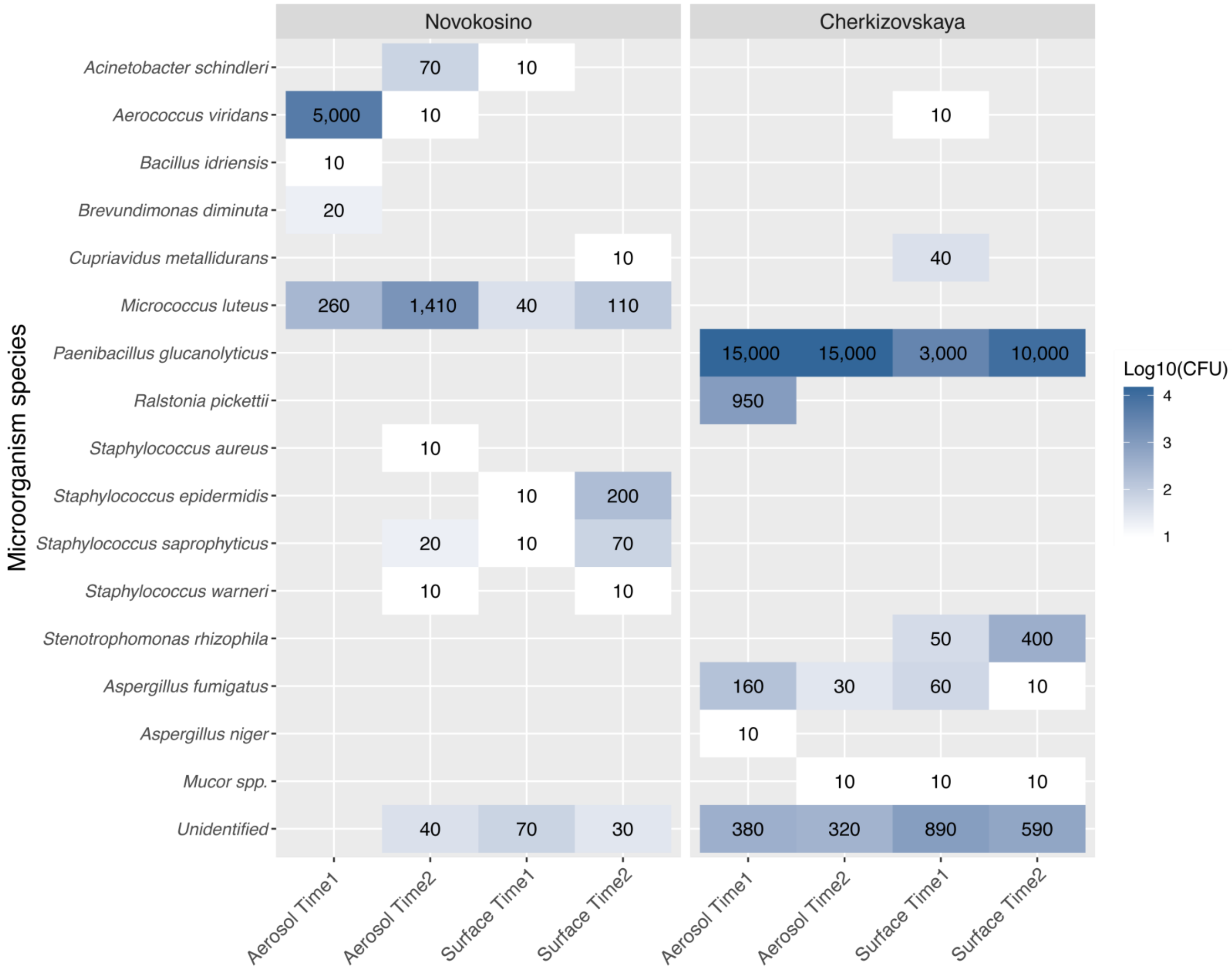

Figure S1. Heat map with identified morphotypes. The numbers indicate reads.

Table S2. Detected antibiotic-resistant isolates. MALDI NRI – (not reliable identification) use MALDI Biotyper.

Station	Sample	Tetracycline	Streptomycin	Ampicillin	Kanamycin	Chloramphenicol	Gentamicin
Novokosino	Aerosol Time1	Abs	*Micrococcus luteus* (2 CFU)	Abs	Abs	Abs	Abs
	Surface Time1	Abs	*Micrococcus luteus* (3 CFU)	Abs	Abs	Abs	Abs
	Aerosol Time2	Abs	*Micrococcus luteus* (9 CFU)	Abs	Abs	*Micrococcus luteus* (4 CFU)	Abs
	Surface Time2	Abs	Abs	Abs	Abs	Abs	Abs
Cherkizovskaya	Aerosol Time1	*Streptomyces albus* (30 CFU)	*Paenibacillus thiaminolyticus* (6 CFU) *Paenibacillus nematophilus* (8 CFU)	Abs	*Paenibacillus macerans* (3 CFU) *Paenibacillus ehimensis* (2 CFU)	*Streptomyces albus* (5 CFU)	Abs
	Surface Time1	*Streptomyces albus* (11 CFU)	*Paenibacillus lacti* (1 CFU) *Paenibacillus cookie* (1 CFU)	Abs	*Paenibacillus thiaminolyticus* (4 CFU)	Abs	Abs
	Aerosol Time2	*Streptomyces albus* (4 CFU)	*Brevibacillus borstelensis* (4 CFU)	Abs	*Brevibacillus borstelensis* (2 CFU)	Abs	Abs
	Surface Time2	*Paenibacillus cookii* (17 CFU)	*Paenibacillus cookie* (7 CFU) *Agromyces mediolanus* (4 CFU) MALDI NRI (4 CFU)	Abs	Abs	Abs	*Sphingobacterium mizutaii* (1 CFU)
